# Effectiveness of long-term cluster training and traditional resistance training in enhancing maximum strength in young adults: a systematic review and meta-analysis

**DOI:** 10.3389/fphys.2025.1568247

**Published:** 2025-04-01

**Authors:** Jiayue Cui, Yin Yu, Yijun Xu, Hongyu Wu

**Affiliations:** ^1^ School of Sports Training, Wuhan Sports University, Wuhan, China; ^2^ Research Center for High-Quality Development of Characteristic Competitive Sports, Wuhan Sports University, Wuhan, China; ^3^ Economics and Management School, Wuhan Sports University, Wuhan, China

**Keywords:** cluster training, training duration, maximum strength, young adults, meta-analysis

## Abstract

**Background:**

It is still unclear whether traditional resistance training (TRT) provides the best or optimal stimulation for increasing maximum strength compared to cluster training (CT).

**Objective:**

This study assessed the long-term impact of cluster training on the augmentation of maximum strength in young adults through the implementation of meta-analysis and further investigation of the factors associated with training duration.

**Method:**

Literature was searched in Web of Science, Pub Med, EBSCOhost, SPORTDiscus, and Google Scholar. After screening, 21 articles and 49 reports were included. Revman 5.4 was used for literature quality evaluation, heterogeneity testing, and data consolidation. Stata 15.1 was used for drawing forest plots, subgroup analysis, taking sensitivity analysis and meta-regression to explore the sources of heterogeneity, creating a funnel plot to evaluate publication bias, quantifying publication bias, and trimming and filling. The original protocol was prospectively registered at the PROSPERO (CRD42024547097).

**Result:**

The random effects meta-analysis results showed significant heterogeneity (I^2^ = 70.7%), SMD = 0.10, 95% confidence interval (CI) [-0.14, 0.33], indicating no difference between CT and TRT in general. However, considering training duration, CT was more effective in 4–8 weeks (SMD = 0.24, 95%CI [0.06, 0.42]), while TRT was better in 9–12 weeks (SMD = −1.54, 95%CI [-3.03, −0.05]). Sub-group analysis found that CT had a better effect on people aged 23 and above (SMD = 0.38, 95% CI [0.11, 0.65]), and there was no significant difference in sex and participant type.

**Conclusion:**

Cluster training (CT) mitigates exercise-induced fatigue more effectively than traditional resistance training (TRT) and enables more efficient maximum strength growth within the initial 8 weeks, however, the converse holds after 9 weeks. For preparation periods of 8 weeks or less, such as a microcycle or a specific stage in block periodization, trainers are advised to adopt CT for enhancing or maintaining maximum strength. This suggests that trainers, when undertaking maximum strength training, whether short-term or long-term, can not only consider CT but also precisely schedule the time-course of resistance training modalities within continuous periodization. Specifically, they can switch to TRT after 8-weeks of CT to achieve more favorable training outcomes.

**Systematic Review Registration:**

PROSPERO

## 1 Introduction

Structured and continuous resistance training (RT) is essential in developing various physical performance characteristics, but progression in resistance training is a dynamic process, it is impossible to improve at the same rate over long-term periods (American College of Sports Medicine, 2009). For progression, the exercise prescription must be altered over time to maintain or advance specific training goals. Meanwhile, different training methods, loads, intensities, durations, and intervals are important factors when designing resistance training plans (McLeod et al., 2024; Li et al., 2024). Depending on the manipulation of these variables can lead to different adaptations and varying magnitudes of increase in strength, power, endurance, and muscle hypertrophy. Traditional resistance training (TRT) is conducted continuously with no rest between repetitions, with inter-set rest ranging from 1 to 5 min (Tufano et al., 2017a). However, it is still being determined whether this method provides the best or optimal stimulation compared to other methods. In addition, in long-term training plans, it is necessary to further consider the focus of the overall objectives during the training phase (Davies et al., 2021). In recent years, many scholars and practitioners have attempted to vary the traditional set structures of TRT (6) to minimize or mitigate the negative impacts of neuromuscular fatigue and generate more significant adaptive changes as much as possible. It is under the guidance of this line of thinking that cluster training (CT) has emerged and developed.

Cluster training, the primary method based on the traditional structures of TRT, adds interval rest or redistributes time within each set of exercises to reduce accumulated fatigue during training and maximize individual repetition efficiency and performance (Zhong et al., 2024; Haff et al., 2008a; Tufano et al., 2016). At present, CT has formed five representative types, namely intra-set rest (IR), inter-repetition rest (IRR), rest-pause (RP), redistribution rest (RR), and equal work-to-rest ratios (EW: R) (Tufano et al., 2017a; Latella et al., 2021). Much research (Tufano et al., 2017a; Hardee et al., 2012; Lawton et al., 2006; Wetmore et al., 2019) has confirmed that CT has a more positive effect on the power output and maintenance of movement speed, especially in acute or short-term effects. On the other hand, there has been extensive discussion in academia regarding the effectiveness of cluster training in enhancing maximum strength. However, previous studies have focused on its overall effects, and the findings appear equivocal. As shown in the study by Zaras et al. (2022), the maximum strength percentage increase was more significant in the inter-repetition rest group than in the TRT group. Meanwhile, Enes et al.'s study (Enes et al., 2021) suggests that the rest-pause method promotes a higher 1RM effect than TRT. Both Oliver et al. (2013) and Samson and Pillai (2018) indicated that shorter but more frequent rest periods allow for more significant improvements in 1RM strength. In contrast, Goto et al. (2005), Hansen et al. (2011), and Zarezadeh-Mehrizi et al. (2013) all emphasized the importance of fatigue during resistance training in inducing strength gains, as they observed more significant increases in maximum strength in groups using TRT rather than CT structures. Referring to the research results of Carneiro et al. (2020), if the training focus is “force,” traditional training may be preferred. If the training focus is “speed,” cluster training may be preferred. To further complicate matters, some studies (Davies et al., 2021; Jukic et al., 2020; Jukic et al., 2021) have shown that cluster training can be equally practical as traditional resistance training in actively inducing muscle and neuromuscular adaptation with less fatigue accumulation. Once training starts, fatigue occurs. Adding intra-set rest has been proven to reduce the adverse effects of performance decline (such as fatigue) within the group (Haff et al., 2003) and ensure the quality of movements during each repetition (Haff et al., 2008b; Moreno et al., 2014), thus being able to withstand greater loads and higher training intensity (Tufano et al., 2017a; Haff et al., 2008a). Besides, although previous meta-analyses (Tufano et al., 2017a; Davies et al., 2021; Jukic et al., 2020; Jukic et al., 2021) did not indicate that cluster training has a better effect on improving maximum strength, they mentioned that cluster training has similar benefits.

In light of the previous findings, this study employs a systematic review and meta-analysis to integrate and analyze the exact effect of long-term cluster training duration (4–12 weeks) on maximum strength growth. Specifically, in the initial training period, CT will be more effective in enhancing maximum strength due to its unique structure (e.g., intra-set rest, inter-repetition rest) that mitigates exercise-induced fatigue. This allows for higher training intensity and repetition quality, thereby promoting neural adaptations and rapid strength gains. Conversely, as training progresses into the later stages, TRT may become more advantageous due to its ability to induce muscle hypertrophy through sustained fatigue accumulation. Muscle hypertrophy, which is more pronounced with longer-term training, contributes significantly to further strength development. Therefore, this study will also explore how to effectively utilize CT within a long-term training period and how to combine it with TRT to optimize the enhancement of strength and performance.

## 2 Methods

### 2.1 Registration of systematic review protocol

A systematic literature review was performed according to the Cochrane Handbook for Systematic Reviews of Interventions guidelines and following the 2020 checklist for the Preferred Reporting Items for Systematic Reviews and Meta-Analyses (PRISMA) (Page et al., 2021). The original protocol was prospectively registered at the PROSPERO (International Prospective Register of Systematic Reviews). The protocol registration (CRD42024547097) occurred after searches were conducted but before screening was completed and data extraction started.

### 2.2 Eligibility criteria

The inclusion criteria for this review included (American College of Sports Medicine, 2009): scholarly publication in the English language (McLeod et al., 2024); randomized comparative studies (RCT) (Li et al., 2024); participants aged 18–35 years old (Tufano et al., 2017a); recruited participants were free of any medical condition or injury (Davies et al., 2021); training intervention group that utilized a cluster set configuration (i.e., intra-set rest, inter-repetition rest, rest redistribution, rest redistribution and rest-pause models) as defined by Tufano et al. (2017a), Zhong et al. (2024) a comparison training intervention group that utilized a traditional set configuration (i.e. continuous repetitions with no intra-set rest strategy) (Haff et al., 2008a); measure or estimate of changes in maximum strength; and (Tufano et al., 2016) intervention length ≥4 weeks in length (i.e. long-enough to detect changes in maximum strength).

### 2.3 Information sources

This study followed the guidelines of a new edition of the Cochrane Handbook for Systematic Reviews of Interventions (Cumpston et al., 2019) and searched for literature in databases such as Web of Science, Pub Med, EBSCOhost, SPORTDiscus, and Google Scholar. The search terms included TS= (“maximal force” OR “maximum force” OR “maximum strength” OR “maximal strength” OR “1RM” OR “1 repetition* maximum”) AND TS= (“cluster training” OR “cluster set*” OR “cluster structure*” OR “cluster group” OR “rest pause” OR “rest redistribution” OR “intra-set rest” OR “equal work-to-rest ratio” OR “inter-repetition rest”) and TX (“cluster training” or “cluster set*” or “cluster structure*” or “cluster g”oup” or “rest pause” or “rest redistribution” or “intra-set rest” or “equal work-to-rest ratio” or “inter-repetition rest”) AND TX (“maximal force” or “maximum force” or “maximum strength” or “maximal strength” or “1RM” or “1 repetition* maximum”) (with the search date ending on 15 January 2025).

### 2.4 Literature search strategy

P (Participant): Non-disabled, injury-free young adults (including athletes, amateur sports enthusiasts, non-professionally trained individuals, and recreational fitness enthusiasts) with no sex or age restrictions.

I (Intervention): Application of cluster training, including any one or a combination of methods such as IR, IRR, RP, EW:R, and RR, with an intervention period of at least 4 weeks. Training can be unilateral or bilateral, with no restrictions.

C (Comparison): Utilization of a randomized controlled trial (RCT) where the experimental group undergoes cluster training (CT) and the control group undergoes traditional resistance training (TRT), ensuring consistency in experimental testing protocols and intervention duration.

O (Outcome): The outcome measures must include the results of 1RM maximal strength tests conducted before and after the experiment.

S (Study): Randomized controlled studies, both published and unpublished, with clearly defined training protocols that meet the inclusion criteria.

### 2.5 Study selection

**FIGURE 1:**
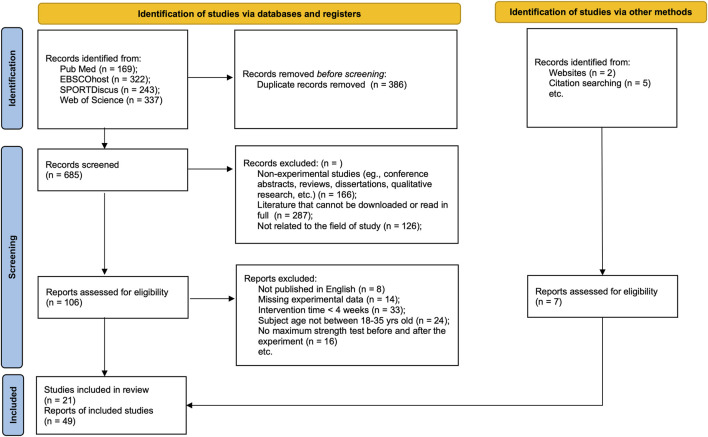
Flow chart of study selection.

Duplicate references were eliminated with the EndNote reference manager, version X9.0.3. Subsequently, two reviewers, Cui and Yu, independently assessed titles and abstracts to ascertain preliminary suitability by the Rayyan systematic review tool. The reviewers were kept unaware of each other’s assessments to prevent bias. The reviewers independently examined the complete texts to ascertain the final inclusion criteria (Figure 1). Any disagreements regarding eligibility at any point were settled via discussion or, if necessary, with the involvement of a third reviewer, Xu.

### 2.6 Data extraction

The data from the included studies were transferred into an Excel spreadsheet, encompassing the following details (American College of Sports Medicine, 2009): study design and identification details (McLeod et al., 2024); adherence and study duration (Li et al., 2024); sample size (Tufano et al., 2017a); participants’ age, body mass, height, sex, strength level, and training background (Davies et al., 2021); specifics on cluster training employed, including methodological aspects (e.g., IR, IRR, RP, EW: R and RR); and (Zhong et al., 2024) means, standard deviations, and raw mean changes with their standard deviations for pre- and post-intervention assessments of the pertinent outcome measures. In cases where data were insufficiently reported, the authors of those studies were contacted by e-mail. Origin software was utilized to extract data from figures when the authors did not provide the necessary data. Two authors (Cui and Yu) independently conducted the data extraction. The coding files were then cross-verified between the authors, with discrepancies settled through discussion and consensus or with the involvement of a third reviewer (Xu).

### 2.7 Statistical analysis

Quantitatively evaluate heterogeneity using I^2^ values, with I^2^ values of 0%, 25%, 50%, and 75% indicating no heterogeneity, mild heterogeneity, moderate heterogeneity, and high heterogeneity, respectively (Buccheri et al., 2018). When heterogeneity is significant (I^2^ ≥ 50), a random effects model is used to merge the data; otherwise, a fixed effects model is used.

Revman 5.4 software was used for literature quality evaluation. When the heterogeneity (I^2^) was high, Stata 15.1 software was used for trimming and filling method (Shi and Lin, 2019) to estimate the impact of publication bias on the stability of meta-analysis results, when P > 0.05, the robustness of the original study was demonstrated. Additionally, subgroup analysis and meta-regression were conducted to explore sources of heterogeneity, with P < 0.05 indicating a significant source of heterogeneity. Sensitivity analysis (Shim et al., 2017) was performed to examine the robustness of individual studies, and a funnel plot was created to evaluate publication bias, using Begg and Egger tests to quantify publication bias; with P < 0.1 as the significance level (Phua et al., 2022). In addition, there was no reason to expect that studies finding no significant difference between different set structures would be less likely to be published than studies reporting a statistically significant difference.

#### 2.7.1 Determination of the effect size

The standardized mean change for each group was calculated as the difference between post-test and pre-test scores, divided by the pre-test standard deviation with an adjustment for small sample bias (Rubio-Aparicio et al., 2018; Andrade, 2020). After that, the standardized mean difference (SMD) with 95% confidence intervals for the difference was calculated between the two standardized mean change values for each study to indicate how much larger (or smaller) the change in the experimental group was (in standard deviation units) when compared to the change in the control group (Andrade, 2020). The following formulas are used for necessary calculations:

$$SMD=C_{\mathrm{Exp}}\left(\frac{M_{post.\;\mathrm{Exp}}-M_{pre.\;\mathrm{Exp}}}{{SD}_{pre.\mathrm{Exp}}}\right)-C_{Con}\left(\frac{M_{post.\;Con}-M_{pre.\;Con}}{{SD}_{pre.Con}}\right)$$

where C (i.e., the correction for small sample bias) is given by:

$$C_{i}=1-\frac{3}{4\left(n_{i}-1\right)-1}$$

The SMD magnitude was interpreted as small (0.20–0.49), moderate (0.50–0.79), or large (>0.80).

#### 2.7.2 Determination of the correlation and variance

No studies reported the pre-to post-intervention correlations required to determine the variance. Therefore, when the authors did not provide correlations upon our request, standard deviations of the pre-to post-intervention change were used to calculate pre-to-post correlations using the following formula (Cumpston et al., 2019):

$${SD}_{Change}={{SD}_{baseline}}^{2}+{{SD}_{post}}^{2}-\left(\sqrt{2\times Corr\times {SD}_{baseline}\times {SD}_{post}}\right)$$

When either the baseline or post-intervention SD is unavailable, then it may be substituted by the other, providing it is reasonable to assume that the intervention does not alter the variability of the outcome measure. Assuming the correlation coefficients from the two intervention groups are reasonably similar, a simple average can be taken as a reasonable measure of the similarity of baseline and final measurements across all individuals in the study. The correlation coefficient can be calculated as:

$$Corr=\frac{{{SD}_{baseline}}^{2}+{{SD}_{final}}^{2}-{{SD}_{change}}^{2}}{2{{\;\times \;SD}_{baseline}}^{2}\times {{SD}_{final}}^{2}}$$

## 3 Results

### 3.1 Search results and study characteristics

After screening, a total of 21 articles and 49 reports were included. A power analysis (Peterson and Foley, 2021) was conducted to determine that the number of included studies is adequate for drawing strong conclusions. Specifically, the included studies comprised 12 articles (57.1%) using intra-set rest method (IR), 4 articles (19.1%) using inter-repetition rest method (IRR), 2 articles (9.5%) using rest redistribution method (RR), 2 articles (9.5%) using rest-pause method (RP), and 1 article (4.8%) using equal work to rest ratio method (E: WR). The experimental data reports are complete, and although 16 articles did not implement blinding, it did not significantly impact the experimental results. There were 583 participants, including 88 professional athletes and 495 amateur-trained people. The inclusion criteria for the study participants were clear, and they could complete the experiment according to the allocation plan. In most studies, the CT and TRT group participants had similar training amounts (280 participants trained by CT, 276 participants trained by TRT, Table 1).

**TABLE 1:** Basic information of included studies.

Study	*N* (male/female)	Age (mean ± SD)	Participants type	CT type	Outcomes	Measure items	Training duration (weeks)
Aminaei et al. (2017)	0/18	18.22 ± 3.02	Karate players	IR	Maximum strength, Explosive power	1RM of squat, 6 jumps up squat movements at 20% of 1RM	9
Aravena-Sagardia et al. (2021)	15/10	19 ± 1.2	College students	IR	Maximum strength, Explosive power	CMJ, 1RM of bench press, military press, parallel squat, and deadlift	8
Arazi et al. (2018)	0/30	18.5 ± 4.2	Volleyball players	IR	Maximum strength, Peak power output	Thigh and arm circumference, vertical jump, 20 m sprint, 4 × 9 m shuttle-run, 1RM back squat, bench press, military press, deadlift	8
Karsten et al. (2021)	18/0	24 ± 4	Trained males	EW:R	Muscular hypertrophy, Maximum strength, Explosive power	CMJ, 1RM bench press, 1RM squat, 50%1RM bench press power, arm and thigh circumference	6
Enes et al. (2021)	28/0	23.41 ± 3.37	Trained males	RP	Maximal strength, Muscular hypertrophy	1RM and MT of the proximal, middle, and distal portions of the lateral thigh	8
Farinas et al. (2021)	23/12	24 ± 6.3	Physically active subjects	IRR	Maximum strength, Muscular hypertrophy, and endurance	1RM load, number of repetitions with the 10RM load (n10RM), total mechanical work with the 10RM load (10RMW), isometric maximal voluntary contraction (MVC)	5
Fariñas et al. (2023)	29/6	23 ± 2	College students	RR	Maximum strength, Muscular endurance	Unilateral knee extension 1RM load for each leg, 10 repetitions with 50% of the 1RM	5
Goto et al. (2005)	26/0	22.7 ± 0.5	College students	IR	Maximum strength, Muscular hypertrophy, Explosive power	1RM shoulder press, 1RM bilateral knee extension, 70%1RM shoulder press, and bilateral knee extension (reps), the CSA of the thigh	12
Hansen et al. (2011)	18/0	26.8 ± 4.5	Elite rugby union players	IR	Maximum strength, Explosive power	1RM back squat and squat jump	8
Korak et al. (2018)	0/13	23.05 ± 3.28	Trained females	RP	Maximum strength	1RM bench press and electromyography (EMG)	4
Mao et al. (2023)	30/0	21.5 ± 2.98	College students	RR	Maximum strength, Muscular hypertrophy, Explosive Power, Muscular endurance, Velocity	Regional muscle thickness, upper and lower-body muscle maximal strength (1RM), mean power output and velocity at 75% 1RM and muscular endurance (repetitions to failure at 70% 1RM)	8
Moghadam et al. (2023)	47/0	26.5 ± 3.9	Active males	IR	Maximum strength, Muscular endurance, Velocity	1RM back squat and chest press, SLJ, linear 20-m sprinting test and 9-m shuttle run test, 60% 1RM back squat (repetitions)	6
Vargas-Molina et al. (2020)	29/0	26.9 ± 8	Trained males	IR	Maximum strength, Explosive power	1RM squat and CMJ	8
Nicholson et al. (2016)	34/0	21.76 ± 2.6	Trained males	IRR	Maximum strength, Explosive power	1RM back squat, vertical jump	6
Oliver et al. (2013)	22/0	25 ± 5	Trained males	IR	Maximum strength, Explosive Power, Muscular hypertrophy	1RM bench press and squat, mean power output (60% 1RM bench press and squat), vertical jump, and less mass	12
Rial-Vazquez et al. (2020)	28/11	23 ± 4	College students	IR	Maximum strength, Explosive power, Velocity	1RM bench press and squat, force, and velocity axis intercept, slope, and estimated maximum power, 10 repetitions of bench press and squat with the 50% 1RM lat pull-down and leg curl exercises	5
Samson and Pillai (2018)	32/0	18–26	Trained males	IR	Maximum strength	1RM bench press, bent over row, shoulder press, back squat, sumo squat, and calf raises	7
Stragier et al. (2019)	15/15	23.84 ± 3.48	Healthy subjects	IR	Maximum strength, Explosive Power, Muscular hypertrophy	1RM load and maximal isometric voluntary contraction (MVC), EMG activity of biceps brachii and brachioradialis, and biceps’ brachii thickness	12
Zaras et al. (2020)	16/0	22.65 ± 5.09	College students	IRR	Maximum strength, Muscular hypertrophy	1RM leg press, isometric leg press RFD, peak force, VL muscle architecture, vastus intermedius (VI) thickness, and quadriceps’ cross-sectional area (CSA)	7
Zaras et al. (2022)	16/0	22.65 ± 5.09	College students	IRR	Maximum strength, Muscular hypertrophy	1RM bench press, upper body rate of force development and isometric peak force, Triceps brachii ultrasonography	7
Zarezadeh-Mehrizi et al. (2013)	24/0	24.68 ± 3.13	Soccer players	IR	Maximum strength, Explosive power	1RM squat, mean power of 6 squat jumps with 30%1RM	10

N, number of participants; *NR*, not reported; *IR*, Intra-set Rest; *EW, R* Equal Work-to-Rest Ratio; *IRR*, Inter-repetition Rest; *RR*, Rest-redistribution; *RP*, Rest-pause; *RM*, repetition maximum; *CMJ*, countermovement jump; *SLJ*, standing long jump.

### 3.2 Risk of bias assessment

**FIGURE 2:**
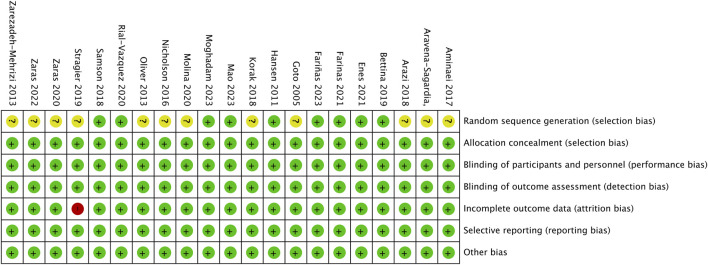
Risk of bias summary of included studies.

The risk of bias for each study was assessed by Cui and independently assessed again by Yu; disagreements were resolved through discussions with Xu. According to the Cochrane risk of bias criteria (Higgins et al., 2019), the included studies were assessed for bias risk (Figure 2) across seven evaluation indicators: random sequence generation, allocation concealment, participant and personnel blinding, outcome assessment blinding, incomplete outcome data, selective reporting, and other sources of bias. The results indicate that 1 study has a high risk of bias, 12 studies have a moderate risk of bias, and the remaining 8 studies are at low risk of bias.

### 3.3 Meta-analysis results of different groups

**FIGURE 3:**
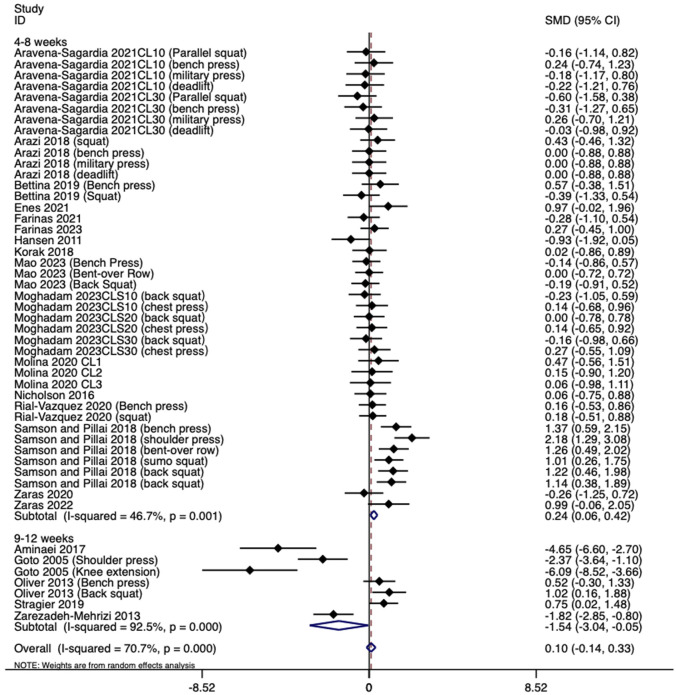
Forest plot of maximum strength increase.

**FIGURE 4:**
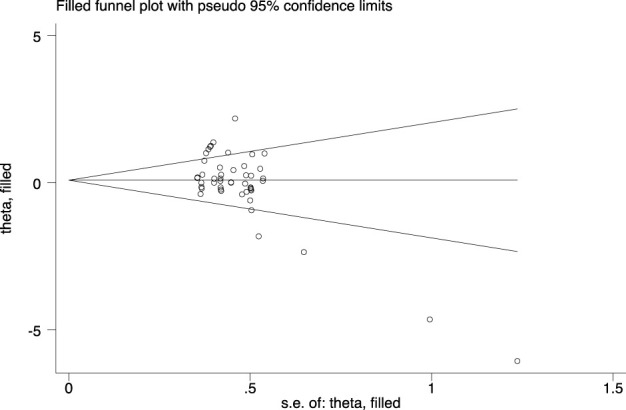
Trim and fill plot of maximum strength outcome.

The meta-analysis results showed significant heterogeneity between studies (I^2^ = 70.7%), so a random effects model was used to obtain SMD = 0.10 and 95% CI [-0.14, 0.33]. The pooled analysis result showed no difference between cluster and traditional training configurations. The merged results intersected with the invalid line (P > 0.05, Figure 3). Although the results of Begg and Egger’s tests (t = −5.00, P < 0.01) suggested the existence of publication bias, the results of the trim and fill method did not supplement virtual literature, and the results did not reverse (P > 0.1, Figure 4). This indicates that although the original studies had relatively high heterogeneity, the publication bias and results were stable, and these studies could be merged for meta-analysis. Meta-regression analysis found that participants type (athletes or non-athletes), training duration, age, and sample size are the sources of heterogeneity (P < 0.01), while CT type and sex are not sources of heterogeneity (P > 0.05).

Among the 21 included studies, there were 16 studies, with 42 reports having an intervention duration of 4–8 weeks and 5 studies with 7 reports having an intervention duration of 9–12 weeks. As shown in Figure 3, the SMD and 95% CI of 4–8 weeks are 0.24 [0.06, 0.42], and the merged result is to the right of the invalid line (P < 0.05). The SMD and 95% CI of 9–12 weeks are −1.54 [−3.03,-0.05], and the merged result is to the left of the invalid line. This indicates that for the maximum strength growth effect, CT has a more significant effect on the maximum strength growth compared to TRT during 4–8 weeks; During 9–12 weeks, traditional training has better results.

In addition, the sub-group analysis (Table 2) revealed that neither the athlete group (SMD = 0.78 [95% CI: −1.65,0.10]; p = 0.081; Z = 1.74, I^2^ = 81.5%) nor the non-athlete group (SMD = 0.22 [95% CI: −0.004, 0.45]; p = 0.055; Z = 1.92, I^2^ = 65.2%) exerted a significant impact. Although the group with 18–23 years (SMD = −0.33 [95% CI: −0.70, 0.05]; p = 0.088; Z = 1.70, I^2^ = 68.9%) had no significant influence, there is a significant difference in the group with age ≥23 years, as its result is to the right of the invalid line (SMD = 0.38 [95% CI: 0.11, 0.65]; p = 0.006; Z = 2.75, I^2^ = 66.1%). As for sex, neither the male group (SMD = 0.18 [95% CI: −0.14, 0.51]; p = 0.274; Z = 1.09, I^2^ = 76.7%) nor the female group (SMD = −0.54 [95% CI: −1.60, 0.52]; p = 0.317; Z = 1.00, I^2^ = 82.1%) nor the mixed group (SMD = 0.07 [95% CI: −0.17, 0.30]; p = 0.587; Z = 0.54, I^2^ = 0%) had significant impact.

**TABLE 2:** Statistics of subgroup meta-analysis results.

	Subgroup		I^2^	Z	P	SMD [95%CI]
Maximum strength	Training Duration	4–8 weeks	46.7%	2.58	0.010*	0.24 [0.06,0.42]
		9–12 weeks	92.5%	2.02	0.043*	−1.54 [-3.03,-0.05]
	Age	18–23 years old	68.9%	1.70	0.088	−0.33 [-0.70,0.05]
		23–35 years old	66.1%	2.75	0.006*	0.38 [0.11,0.65]
	Participant Type	Athlete	81.5%	1.74	0.081	−0.78 [-1.65,0.10]
		Non-athlete	65.2%	1.92	0.055	0.22 [-0.004,0.45]
	Participant Sex	Male	76.7%	1.09	0.274	0.18 [-0.14,0.51]
		Female	82.1%	1.00	0.317	−0.54 [-1.60,0.52]
		Mixed	0%	0.54	0.587	0.07 [-0.17,0.30]
	Overall		70.7%	0.81	0.420	0.10 [-014,0.33]

*Significant difference, P < 0.05.

## 4 Discussion

Although there is no significant difference in the overall effect of improving maximum strength between the two training modes, cluster training can enable more efficient maximum strength exercises in the first 8 weeks compared with traditional resistance training, while the effect is the opposite after 9 weeks. As is well known, neural factors play an important role in muscle strength gains, neural adaptations are the predominant mechanism for increases in muscular strength in the early phases (first 6–8 weeks) of resistance training (Siddique et al., 2020). The lower the extent of exercise-induced fatigue, the higher the excitability of motor neurons (Alix-Fages et al., 2022), the faster the firing rate (Jones et al., 2023) (sometimes doublet firing and synchronizatio occurs), the more activation of motor units (Contessa et al., 2018), and the greater the force output of muscle strength, which can provide greater training stimulation. On the contrary, central and peripheral motor unit features are altered following exercise-induced fatigue (Jones et al., 2023) and fail to effectively stimulate the nervous system to enhance the force of muscle contraction. Cluster training, to a certain extent, reduces the adverse effects of exercise-induced fatigue, just as research by Haff et al. (2008a), adding about 15 s of recovery within the set, the force attenuation caused by fatigue can be restored by half, and the ability to generate force can be restored to about 79.7% ± 2.3% of the initial ability, which helps to cope with larger loads during training, reaching higher thresholds and recruiting and exercising more exercise units. In addition, Škarabot et al. (2021) have found that after resistance training, the threshold of activated neurons can be reduced, and the firing rate can be increased, allowing subsequent stimuli to recruit more motor units to participate in muscle activity and output higher strength. And the above are precisely the characteristics and advantages of cluster training mode.

Thus, this result may be because cluster training accumulates less fatigue during training. The performance results of the mechanical force values of movements in cluster set training are better than those in traditional set training (i.e., with a higher average output of movement force values or a smaller decline range of movement force values), which can help muscles adapt to larger loads in the early stage of resistance training.

The key factors in developing muscle strength are the combined effects of improving neural coordination ability and increasing the cross-sectional area (CSA) of muscle fibers (muscle hypertrophy) (Weakley et al., 2023). The stimulation must cause the neuromuscular system to reach a sufficient degree of fatigue to increase the duration of muscle tension, enhance the mobilization of motor units, and induce sufficient stimulation of the body’s metabolic and hormonal responses to achieve the optimal development of muscle strength. Therefore, from the perspective of development, many studies (Mayo et al., 2014; Tufano et al., 2017b) have also pointed out that cluster set training reduces the fatigue stimulation of metabolism, nerves, and hormonal responses when conducting resistance training aimed at maximum strength, especially when the load intensity, the number of exercise sets and repetitions, and the rest time are the same, it is more appropriate to adopt the traditional set structure. However, there has also been some new understanding of this issue in recent years. Some scholars have pointed out that cluster set training reduces neuromuscular fatigue (Páez-Maldonado et al., 2024); it also provides the possibility of increasing the load stimulation of training intensity and volume; volume has been shown to affect neural, hypertrophic, metabolic, and hormonal responses and subsequent adaptations to resistant training (American College of Sports Medicine, 2009), which may contribute to the growth of strength qualities. For example, Tufano et al. (2017b) proposed through experiments that compared with the traditional set structure, the cluster set structure (intra-set rest structure) uses a more significant load; although it causes a certain decline in the average speed and peak speed, it ultimately completes a greater total work and prolongs the duration of muscle tension, thus being beneficial to the improvement of maximum muscle strength.

Since there is also an apparent positive correlation between muscle strength levels and training volume, increasing training volume plays a crucial role in strength development. Existing research (Saeterbakken et al., 2024) has confirmed that under a given number of repetitions of an action, a training program that achieves greater work output is more conducive to skeletal muscle hypertrophy, which in turn is beneficial to the development of strength qualities; meanwhile, the increase in muscle tension time will lead to an improvement in muscle activity and an increase in neuromuscular fatigue, thus also promoting the development of skeletal muscle hypertrophy (Stragier et al., 2019). Based precisely on these physiological grounds, the above-mentioned multiple studies have also pointed out that, compared with traditional training, the cluster set structure can increase the training load and achieve an increase in exercise volume without significantly reducing the peak power and speed of the movement (compared with the traditional set structure), and even achieve a simultaneous increase in load intensity and exercise volume, completing greater total work output. Further, using multiple training loads appears to be most beneficial for long-term progression in muscular strength (Suchomel et al., 2018). Therefore, it can be used as an alternative method to traditional resistance training oriented toward strength.

However, it is important to note that our meta-analysis revealed significant heterogeneity (I^2^ = 70.7%) among the studies included, indicating substantial variability in the effects of CT and TRT on maximum strength. We identified participant type, training duration, age, and sample size as the primary sources of heterogeneity through subgroup analysis and meta-regression. Firstly, significant differences in training background and physiological adaptability between athletes and non-athletes may lead to varying responses to training methods (Suchomel et al., 2018). Secondly, differences in training duration may influence training outcomes. Short-term training may primarily enhance strength through neural adaptations, while long-term training may rely more on muscle hypertrophy (American College of Sports Medicine, 2009). Additionally, age differences may cause fluctuations in results. Younger participants may exhibit greater muscle plasticity. Finally, differences in sample size may affect the reliability of results. Small-sample studies may overestimate or underestimate effect sizes, while large-sample studies generally yield more stable results (Andrade, 2020). Therefore, future research should control for these factors or conduct more in-depth studies targeting specific groups to reduce heterogeneity and improve the reliability of results.

It is important to acknowledge the limitations of our meta-analysis. One significant limitation is the presence of publication bias, which was found to be statistically significant (P < 0.05). This suggests that studies with non-significant or negative results may be underrepresented in the literature, potentially skewing our findings towards more favorable outcomes for both CT and TRT. Given this bias, our conclusions should be interpreted with caution. The potential overrepresentation of positive results may lead to an overestimation of the effectiveness of both CT and TRT.

Our meta-analysis provides valuable insights into the comparative effectiveness of long-term cluster training (CT) and traditional resistance training (TRT) in enhancing maximum strength in young adults. The results indicate that CT is more effective in shorter training periods (4–8 weeks), while TRT shows greater benefits in longer training durations (9–12 weeks). This suggests that practitioners should consider the specific training goals and timeframes when selecting between CT and TRT. For a preparation period is 8 weeks or less (such as microcycle or particular stage in block periodization), practitioners are better suited to adopt CT to enhance or maintain maximum strength levels, such as during the NBA playoffs. Conversely, for longer-term training programs (i.e., macrocycle) aimed at muscle hypertrophy and sustained strength gains, TRT may be more suitable. In addition, trainers can shorten training periodization, making training more efficient. For example, after athletes reach their maximum strength goal in advance, they can start other aspects of training earlier. In the increasingly dense sports competition schedules, shortening the training duration for coaches and athletes is valuable.

Based on the above conclusions, this implies that trainers can not only consider using cluster training when conducting maximum strength training but can also accurately schedule the training time-course of resistance training modes within a continuous periodization training (such as switching to TRT after 8-weeks CT for further training) to achieve higher training outcomes. The sub-group analysis results also found that cluster training has a better maximum strength growth effect on people aged 23–35. At the same time, there is no significant difference for people aged 18–23. The intra-set rest brings greater benefits to people aged 23–35. The reason may be that their nervous and musculoskeletal systems are more developed. Although it is not yet known whether their training experience is longer, a broader neurophysiological background, better neural motor control, coordination, and cognitive perception abilities are beneficial for mastering training methods, thus having a specific positive impact on training effectiveness.

## 5 Conclusion

During the 4–8 weeks of training, cluster training (CT) has a more significant effect on maximum strength growth compared to traditional resistance training (TRT). During the training period of 9–12 weeks, traditional training is more beneficial to the growth of maximum strength than cluster training, and as the training duration extends, the beneficial effect of cluster training on maximum strength gradually weakens and tends to stabilize, while the beneficial effect of traditional training gradually increases. This is related to the fact that the strength increase in the early stage of resistance training is mainly from nerve adaptation, and the strength increase in further long-term training is more inclined to the effect of muscle hypertrophy. Regarding age, cluster training has a better effect on maximum strength growth for people aged 23–35, while there is no significant difference for people aged 18–23. Neither sexes nor participant types (athletes or non-athletes) show significant differences with different training methods.

## 6 Practical applications

In previous studies, cluster training was often discussed as a method to minimize fatigue and emphasizes exercise quality. However, this study found that cluster training can significantly increase maximum strength, which may further expand its application value in sports training practice. Additionally, cluster training can achieve greater intensity and volume, but this often comes at the cost of prolonging the training time. Notwithstanding the number of questions remaining unanswered regarding the underlying physiological differences between exercise methods, the waning effectiveness of neural adaptations indicates that 8 weeks of cluster training may be a point where adaptations of maximum strength growth have been optimally reached. As such, coupling the impact of shorter-duration cluster training (4–8 weeks) on overall effectiveness, i.e., implementing intermittent and varying intensities of training through a periodization of the training program, may maximize the overall impact that cluster training can have. This approach could enhance the benefits of cluster training by strategically combining different training intensities and durations, leading to more significant improvements in overall performance and strength gains. Therefore, it can be an alternative method to traditional resistance training oriented towards maximum strength. It may also achieve higher training results by switching from 4 to 8 weeks cluster training to traditional training mode for further training.

## 7 Limitation

The testing methods in the literature, such as the maximum strength test, are inconsistent. Some studies use the bench press, whereas others use the back squat. Future research should focus on studies with consistent testing methods and consider how subjects adapt to equipment and exert force, as these factors can affect test accuracy. Additionally, differences across studies (e.g., types of cluster training, participant demographics, training frequency) need further exploration. In the current research on cluster training, compared with the common inter-repetition rest and intra-set rest, there are few studies on the equal work-to-rest ratio, and only one study using the EW:R is included in this study. Additionally, future research should consider incorporating “maximal or maximum power” into the search strategy to capture a more comprehensive body of literature related to the effects of CT on both strength and power and investigate the effects of CT on diverse populations, including minors, the elderly, and individuals with disabilities.

## Data Availability

The original contributions presented in the study are included in the article/supplementary material, further inquiries can be directed to the corresponding author.
